# Multiphoton imaging reveals that nanosecond pulsed electric fields collapse tumor and normal vascular perfusion in human glioblastoma xenografts

**DOI:** 10.1038/srep34443

**Published:** 2016-10-04

**Authors:** Sylvia M. Bardet, Lynn Carr, Malak Soueid, Delia Arnaud-Cormos, Philippe Leveque, Rodney P. O’Connor

**Affiliations:** 1 XLIM Research Institute, UMR CNRS No 7252, University of Limoges, Faculty of Science and Techniques, 123 Avenue Albert Thomas, 87060 Limoges, France

## Abstract

Despite the biomedical advances of the last century, many cancers including glioblastoma are still resistant to existing therapies leaving patients with poor prognoses. Nanosecond pulsed electric fields (nsPEF) are a promising technology for the treatment of cancer that have thus far been evaluated *in vitro* and in superficial malignancies. In this paper, we develop a tumor organoid model of glioblastoma and apply intravital multiphoton microscopy to assess their response to nsPEFs. We demonstrate for the first time that a single 10 ns, high voltage electric pulse (35–45 kV/cm), collapses the perfusion of neovasculature, and also alters the diameter of capillaries and larger vessels in normal tissue. These results contribute to the fundamental understanding of nsPEF effects in complex tissue environments, and confirm the potential of nsPEFs to disrupt the microenvironment of solid tumors such as glioblastoma.

The brain cancer glioblastoma multiforme (GBM) is incurable and leaves patients with an average survival of approximately 14.6 months after initial diagnosis, despite multimodal treatment with surgery, radiotherapy and chemotherapy^1^. Emerging bioelectric therapies such as electrochemotherapy, electrogenetherapy^2^,^3^ and irreversible electroporation^4^ have yet to be applied clinically on human cancers of the brain, but preclinical studies have shown the potential of these electroporation-based technologies in neuro-oncology^5^,^6^.

Nanosecond pulsed electric fields (nsPEFs) have shown great promise in treating cancer^7^,^8^. At present there have been no investigations of their effects on human glioma or malignancies of the brain. It remains to be determined whether glioblastoma are sensitive to nsPEFs *in vivo*, and whether it is possible to electrically treat a tumor in the brain without damaging surrounding neurons, glia and vasculature. *In vivo* models are therefore needed that permit the study of complex tissue reactions to nsPEFs in the intact brain.

It is important to first consider the more general issue of whether nsPEFs can be used on highly vascularized tumors like glioma. Thus far, nsPEFs have shown significant promise in the treatment of superficial cancers like melanoma^8^,^9^,^10^, papilloma and squamous cell carcinoma^11^. Evidence for the potential of nsPEF to target deep tissue, solid tumors is also encouraging from preclinical animal experiments performed in models of hepatocellular carcinoma^12^,^13^ and breast cancer^14^. A gap currently exists between mechanistic *in vitro* studies on cultured cancer cells and treatment responses observed in animal or human trials of nsPEF therapies.

Here we present a method to assess nsPEF effects on a 3D glioblastoma tumor xenograft grown and vascularized in the avian chorioallantoic membrane (CAM). This model has previously been used to explore angiogenesis phenomena^15^,^16^, to assess nanoparticle uptake kinetics^17^,^18^, and has many other applications in bioengineering^19^. We have combined multiphoton imaging with the CAM model using the quail egg and developed a well characterized exposure system to apply nsPEF to this vascularized tumor organoid. The influence of nsPEFs on tumor vasculature was investigated using multiphoton intravital imaging to demonstrate that a single nsPEF pulse was sufficient to collapse tumor perfusion. In the present work, we have focused primarily on the short term effects of a single pulse on neo- and endovascular structures, rather than long term treatment effects on tumors. The resulting quail CAM approach is a cost-effective and efficient preparation for screening the effects of nsPEF on a wide number of human cancer types, allowing the observation of tumor microenvironment and cell signaling responses to nsPEFs under intravital conditions.

## Results

### Growth of 3D vascularized glioblastoma organoids in the CAM model

**Figure 1:**
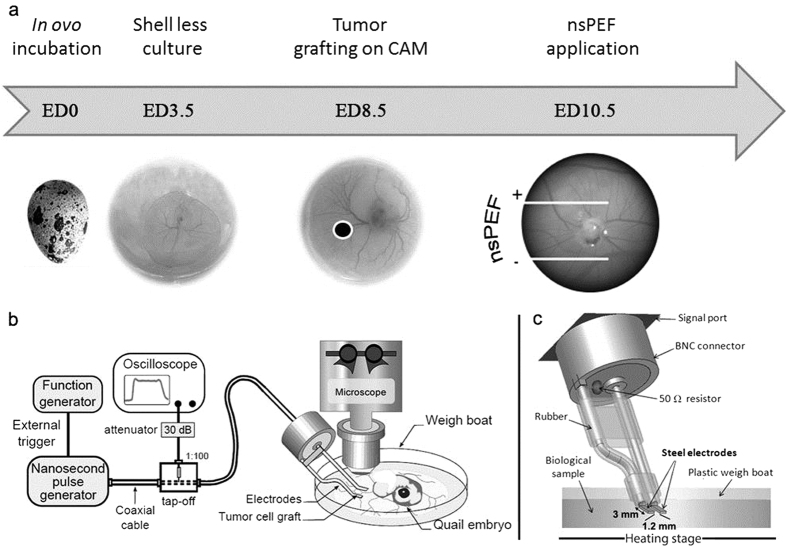
The quail chorioallantoic membrane (CAM) tumor organoid cultivation system for multiphoton imaging of nsPEF effects. (**a**) Schematic view of the *ex ovo* cultivation procedure using quail embryo for the CAM assay. Eggs were opened at embryonic day (ED) 3.5, allowing development in a shell less manner prior to grafting. *In vitro* pre-cultivated pellets of tumor cells were then deposited on the CAM at ED8.5 and vascularization was observed 48 h after (ED10.5), when electromagnetic field applications and imaging were carried out, (**b)** Nanosecond pulse generator, measurement device (oscilloscope) and microscope stage, (**c)** The modeled structure of the electrode-based delivery system.

**Figure 2:**
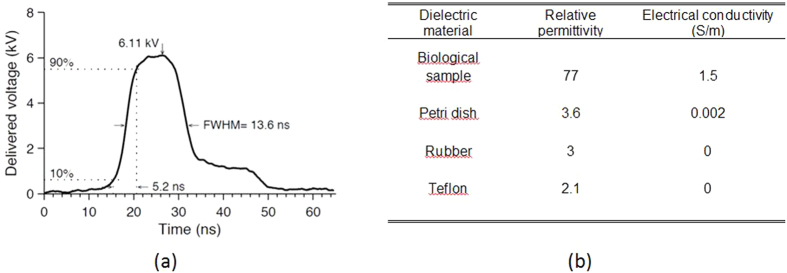
Measured pulse characteristics delivered by the 10 ns PEF generator. (**a**) The peak amplitude and shape of the 6.1 kV electric pulse had a rise time of 5.2 ns and full width at half magnitude (FWHM) of 13.6 ns, (**b**) The relative permittivities and electrical conductivities of the dielectric materials used in all numerical simulations.

Human glioblastoma tumor organoids were grown in shell-less quail egg chorioallantoic membranes (CAM) for multiphoton intravital imaging investigations. Cultured glioblastoma cells (U87-MG) stably expressing green fluorescent protein (GFP) were grafted into developing quail eggs in a developmental window (8.5 embryonic day) when the CAM exhibited a vasoproliferative response that facilitated the growth of the tumor (Fig. 1a)^20^. This assay used the developing quail embryo as a host to generate human tumor organoids at its periphery. The method permitted the growth of fluorescent, spheroidal, millimeter size, vascularized tumors that could be treated with nsPEFs during intravital multiphoton microscopy sessions (Fig. 2a,b). The vasculature of these structures was visualized by injection of a branched polysaccharide-conjugated fluorescent dye (Rhodamine B-dextran 70 K) into the microcirculation of the quail CAM.

### Electromagnetic dosimetry

**Figure 3:**
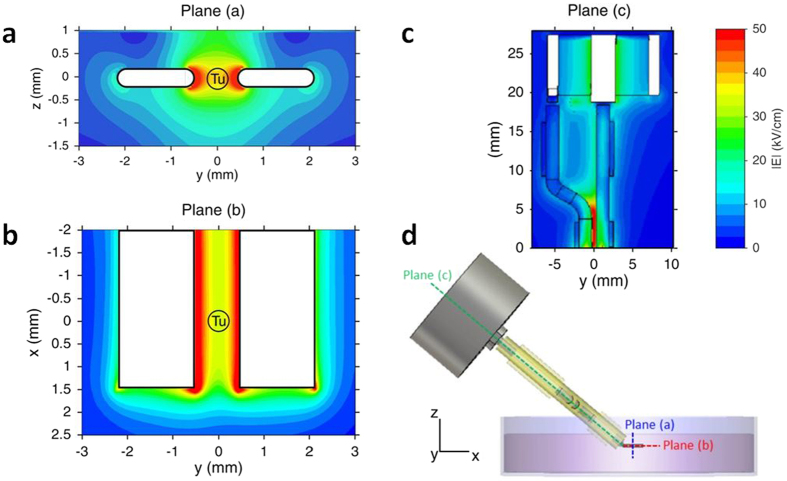
Spatial distribution of the electric field intensity achieved by the delivery system for an incident pulse of 6.1 kV. **a**) Shows the electric field in the 2D vertical plane perpendicular to the electrode tip, (**b**) represents the electric field in the 2D plane between the electrode tips, (**c**) shows the electric field distribution in the 2D plane along the electrodes and the connector. (**d**) Is a side view of the electrode system as orientated during CAM experiments. Tu = Tumor.

A bipolar electrode-based exposure system delivered nsPEFs to the CAM surface on an angle that allowed access to the sample under the microscope objective (Fig. 2b,c). In order to assess the dosimetry of the nsPEF delivery device, numerical simulations were performed to estimate the electric field delivered to the tumors and the CAM. The distribution of the electric field was considered at the macroscopic scale at the electrode level. Figure 3 shows the electric field distribution in the exposure area between electrodes, as obtained using Finite Difference Time Domain (FDTD) numerical methods. The maximum magnitude of the delivered voltage set in this simulation was 6.1 kV, as this was applied in all experiments using the nsPEF generator. Figure 3(a,b) show that the electric field was relatively homogeneous in the central region of the gap between the two electrodes. The amplitude of the electric field was therefore in the range of 35–45 kV/cm.

### Multiphoton imaging reveals the complexity of neovasculature in glioblastoma organoids

Two-photon excitation microscopy (2 PM) of fluorescent U87 tumors with vasculature labeled with Rhodamine B-dextran showed the development of complex networks of neovasculature 1–2 days after the grafting of cells. The endogenous vessels of the CAM were organized in a superficial planar layer of ~200 μm thick and thus easily distinguished from tumor organoid volumes that were visualized using time-lapse 2 PM. Normal blood circulation was observed following the injection of fluorescent dyes and could be followed in CAM up to 24 h after they were returned to the incubator.

**Figure 4:**
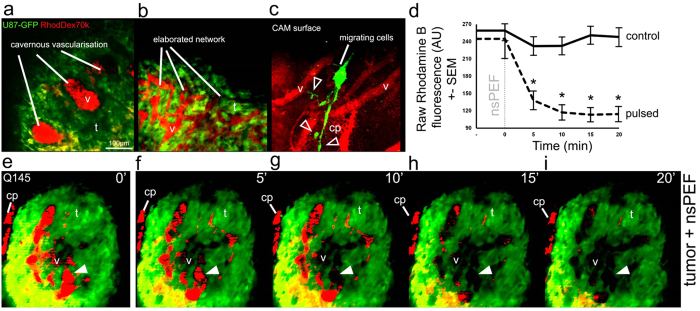
Application of nanosecond pulsed electric fields affects perfusion in U87 xenografts. Intravascular Rhodamine B-dextran and GFP-U87 cells grafted on CAM were observed with multiphoton microscopy, examples of complex vasculature networks are shown on (**a,b**) Migrating cells were occasionally seen on the CAM surface along blood vessels at the periphery of the tumor, white triangle outlines show an example of the extending processes of migrating U87 cells (**c**). Measurement of neovascular fluorescence intensity (Rhodamine B) in tumors is represented in graph (**d**) over time before and after treatment (n = 2 for control, n = 6 for treatment). Asterisks indicate significant differences between pulsed samples and controls over time (p < 0.05). An example of the nsPEF effect on tumor vasculature fluorescence over time is shown for a single focal plane over 5 successive time points, and the solid white triangles indicate an example site in the tumor lumen where fluorescence signal from perfusion (red: Rhodamine B) is clearly lost (**e–i**). AU = Arbitrary Unit, CAM = chorio-allantoic membrane, GFP = Green Fluorescent Protein, RhodDex70 k = Rhodamine B-dextran 70 k, v = vessels, t = tumor mass, cp = capillary plexus. Scale bar in (**a**) = 100 μm applies to all images.

Approximately 75% (129/172) of U87-MG grafts implanted into quail CAM developed into vascularized tumor organoids by embryonic day 10. A subset of tumors (20/172) less than 1 mm in diameter was chosen for imaging experiments as they fit within the dimensions of the exposure system (1.2 mm) and the field of view of the objective (500 μm^2^) (Fig. 1b). As shown in Fig. 4, the architecture of neovascularization was variable between tumors, ranging from sparse, large cavernous vessels (Fig. 4a, Supplementary Video S9) to multiple small vessels organized in complex reticula (Fig. 4b, Supplementary Video S10). Newly formed tumor vasculature was situated above the CAM surface and was therefore easily discriminated from the endogenous vessels (Supplementary Video S8). CAM viability was verified by visualizing blood flow with widefield fluorescence microscopy before 2 PM experiments commenced. Interestingly, U87-MG cells were occasionally observed migrating away from tumor organoids, extending processes (Fig. 4c, arrowheads, Supplementary Video S11) and infiltrating into the CAM along the abluminal surface of blood vessels, as previously reported in rodent xenograft models^21^,^22^.

### Delivery of a single nsPEF disrupts tumor vascular perfusion

The relative change in 3D rendered tumor vasculature was measured from time-lapse Z-stacks taken at 5 min intervals before and following treatment with nsPEFs (Fig. 4d). An example treatment response of a tumor (graft Q145) exhibiting high vascular irrigation (v) and some peripheral capillary plexus (cp) is shown in Fig. 4e–i. The application of a single nanosecond pulse strongly impacted tumor perfusion within 5 min and continued to decrease progressively until it reached 47% of the initial fluorescence intensity at 20 min (black dotted line in Fig. 4d). A two-way repeated measures analysis of variance (ANOVA) was used to test the combined effect of time and nsPEF treatment. The change in fluorescence in the treated tumors over time was statistically significant (interaction effect F_[1.49,86.36]_ = 8.68, p < 0.01). Bonferroni corrected post-hoc tests indicated neovascular fluorescence (Rhodamine B-dextran) in nsPEF treated tumors (n = 6) was significantly (p < 0.05) reduced at 5, 10, 15 and 20 min compared to controls (n = 2), as indicated by asterisks in Fig. 4d. Unfortunately, multiday imaging sessions could not be carried out due to the poor viability of the CAM system after repeated Rhodamine B-dextran injections.

### nsPEFs also affect endogenous vascular flow in the CAM

**Figure 5:**
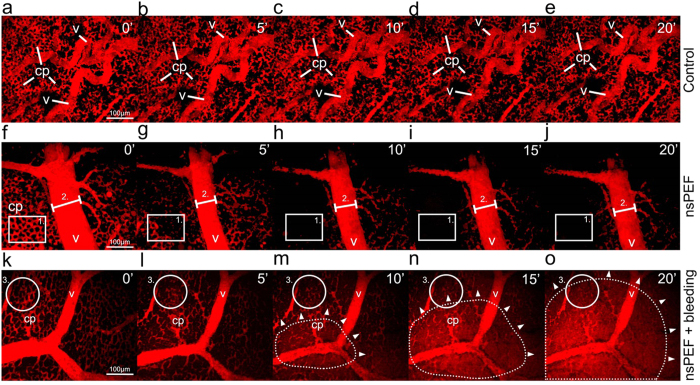
Application of nanosecond pulsed electric fields also affects perfusion in endogenous CAM vascular network. Rhodamine B-dextran was injected intravascularly in the CAM for visualization with multiphoton microscopy, allowing the observation of capillaries (cp) and vessels (v). Examples of vascular fluorescence over time are shown for a single focal plane over 5 successive time points in a control CAM (**a–e**), nsPEF pulsed CAM at t = 0 min without (**f–j**) and with subsequent bleeding (**k–o**). Rectangle zone (**1**) shown in (**f–j**) represents an example of capillary network, while fiducial line (2) shows the diameter of a large vessel. The solid outline (3) on k-o shows an example zone in the capillary bed, and in m-o the dotted outline with white triangles shows the expanding extravasation in the field of view over time following nsPEF treatment. Scale bars in (**a,f,k**) = 100 μm apply to all images.

**Figure 6:**
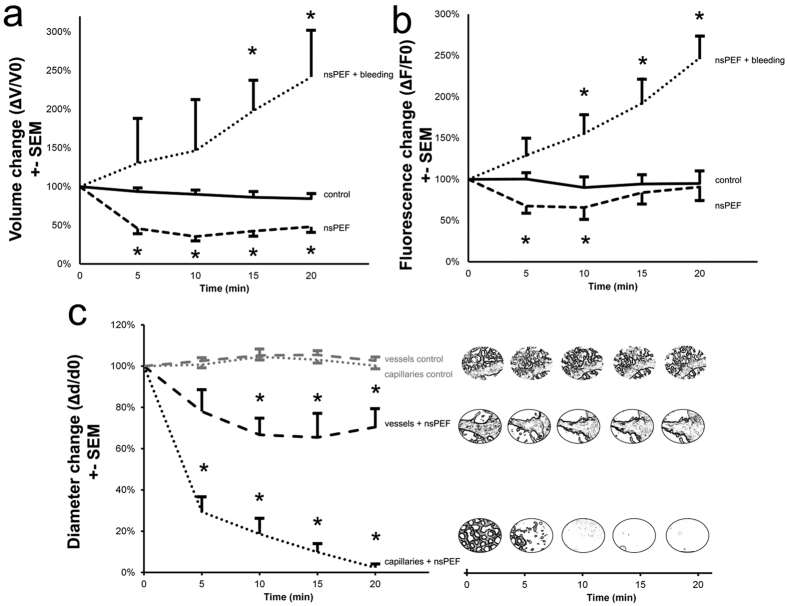
nsPEF treatment influences vasculature in the endogenous CAM vessels. A significant change in volume and fluorescence intensity over time was observed in CAM vessels exposed to nsPEF: **(a)** ΔV/V0 and **(b)** ΔF/F0 mean + standard error of the mean (SEM) of control group (solid line, n = 5), nsPEF group w/o bleeding (dashed line, n = 17) and nsPEF group + bleeding (dotted line, n = 6). **(c)** Vessel diameters were measured from average Z-stacks: the change in diameter is shown as Δd/d0 mean + standard error of the mean (SEM) for the control (grey dotted: capillaries and dashed line: vessels) and pulsed group (black dotted: capillaries+nsPEF and dashed line: vessels+nsPEF). Panels on the right show the morphologic change of the treated or non-treated zone over time. Asterisks indicate significant differences between pulsed samples and controls (p < 0.05).

We next set out to determine whether the application of nsPEFs had any specificity to disrupt tumor neovasculature, or whether the perfusion of endogenous vessels in non-grafted CAMs was similarly affected. The vasculature of the CAM was visualized following intravenous injection of Rhodamine B-dextran 70 K and the fluorescence intensity, volume and diameter of capillaries and larger vessels were measured (Figs 5 and 6, Supplementary Videos S1 to S6). A total of 28 independent CAM samples were studied (n = 5 controls, n = 23 nsPEF treated). The vascular network of the CAM was comprised of large vessels (v) and intricate capillary beds (cp). We observed a bimodal response following the application of a single nsPEF that occurred as changes to blood flow with or without extravasation. We sorted these experimental groups into 2 response subsets (nsPEF+bleeding vs. nsPEF w/o bleeding) as they could be visually and statistically discriminated (One-way ANOVA with Bonferroni post-hoc (F_[1,18.15]_ = , p < 0.005 at t = 20 min) (Fig. 5f–j and Fig. 6a,b for nsPEF w/o bleeding, Fig. 5k–o and Fig. 6a,b for nsPEF+bleeding). Vascular fluorescence in the control group was stable (Fig. 6b, black continuous line), with a less than 4% change in volume over 5 min intervals, and no significant change in fluorescence intensity (F_[1,23]_ = 0.895, p = 0.354). Average Z-projections represented on Fig. 5a–e show different time points of a CAM without nsPEF treatment, demonstrating the richly irrigated capillary plexus (cp) and vessels (v) of different size.

Delivery of a single 10 ns pulse strongly influenced circulation in vessels in the CAM, leading to a significant decrease in blood volume (main nsPEF effect F_[1,18]_ = 15.19, p = 0.001) in the nsPEF treated samples w/o bleeding. As shown in Fig. 6a, the decrease in volume was statistically significant at 5, 10, 15 and 20 min (p < 0.05) as compared to unpulsed controls. The fluorescence intensity of vasculature also showed a similar trend (Fig. 6b, at t = 5 min, 68%) but with a slow recuperation (t = 20 min, 91%), as seen in Fig. 6b. In this case, only the decrease in fluorescence at 5 and 10 min were statistically significant compared to controls (respectively p = 0.001 and p = 0.04).

In the nsPEF+bleeding group (Fig. 6a,b), the volume as well the fluorescence intensity of blood progressively increased by a factor of 2.5 after nsPEF treatment (respectively 242% and 247% at t = 20 min), suggesting a consistent extravasation of dye. In the bleeding group, neither the changes in volume nor the changes in fluorescence intensity were normally distributed and were therefore analyzed separately by non-parametric statistics. The increase in volume in nsPEF+bleeding group was significantly higher than controls at 15 min (χ^2^ = 6.53, p = 0.01) and 20 min (χ^2^ = 6.81, p = 0.009). The increase in fluorescence intensity in nsPEF+bleeding group was also significantly higher than controls at 10 min (χ^2^ = 5.63, p = 0.017), 15 min (χ^2^ = 7.5, p = 0.006) and 20 min (χ^2^ = 6.82, p = 0.009).

The two types of responses, namely decreases in vascular perfusion and decreases with bleeding, could be easily distinguished visually in real-time following nsPEF treatment (Supplementary Videos S3 versus S5). This can be seen clearly in Fig. 5f–j, where the background remains dark, whilst the extravascular space progressively shows an increase in red fluorescence in Fig. 5m–o. Dye leakage appeared to occur throughout the field of view, but could often be observed as an enlarging zone (dotted zone) expanding out from a central region (white arrowheads), as shown in Fig. 5m–o.

In both nsPEF response types, the most significant change occurred in the capillary plexus (Fig. 5f–j zone 1 and k-o zone 3) and was visually similar to the drop in fluorescence perfusion observed in treated tumor organoids (Supplementary Videos S3). In larger vessels of the CAM (v), nsPEF treatment narrowed vessel size (Fig. 5f–j, fiducial marker 2). To quantify this effect, the diameters of capillaries (15.40 μm ± 7.84) and larger vessels (36.69 μm ± 14.96) were measured (Fig. 6c). Vascular diameter was highly stable in the control samples not exposed to nsPEFs (Fig. 6c grey lines, dot: capillaries, dash: vessels and b). In contrast, the application of a single 10 ns pulse drastically reduced capillary diameter (Fig. 6c black dot, a reduction to 2% ± 4 of initial diameter). Analysis by repeated measure 2-way mixed-model ANOVA revealed a significant main effect of nsPEF treatment on capillary diameter (F_[1,25]_ = 301.19, p = 1.28e^−15^) and a significant interaction effect of the treatment over time (F_[3,75]_ = 2.84, p = 0.043). The change in capillary diameter was statistically greater at 5, 10, 15 and 20 min (p < 0.05) as compared to controls, shown by asterisks (Fig. 6c, dotted curve, capillaries+nsPEF). The change in large vessel diameter was also statistically greater in nsPEF treated samples as compared to controls at 10, 15 and 20 min (F_[1,19]_ = 17.32, p = 5.3e^−4^) (Fig. 6c black dashed curve, vessels+nsPEF, asterisks p < 0.05).

The nsPEF response therefore was greater in the small disordered vessels of the tumor organoids, and also in the capillaries of endogenous vessels of the CAM. As shown in Supplementary Fig. 1, nsPEF treatment decreased large vessel size at 5 min, leading to a clear zone without blood flow (SFig. 1b, circular zone 1). At 12 h, large vessel perfusion returned but not to the majority of capillaries in the central zone of the exposure site (SFig. 1c, circular zone 2 compared to zone 3).

The dose-response relationship between electric field intensity of a single nsPEF was investigated on the vascular contraction response of large vessels (Supplementary Fig. 2). A total of 18 independent CAM samples were injected with Rhodamine B-dextran 70 k and treated with a single nsPEF at a range of electric field intensities including 0 kV/cm (control condition with the same placement of electrodes, n = 4), 10.6 kV/cm (n = 2), 16.5 kV/cm (n = 2), 22.5 kV/cm (n = 3), 34 kV/cm (n = 3) and 44 kV/cm (n = 4). Vessel diameter was measured and the peak decrease in vessel diameter followed a sigmoidal trend with increasing electric field intensity, as shown in Supplementary Fig. 2 by the dose-response curve fit (R-Square = 0.99). The dose-response curve indicated that a single 10 ns pulse with an electric field of 22.8 kV/cm +/−5.2 was required to elicit a 50% change in vessel diameter (EC50).

## Discussion

In this study, we have leveraged the power of multiphoton microscopy and a 3D glioblastoma organoid model to demonstrate that a single 10 nanosecond electric pulse was sufficient to disrupt tumor vascular perfusion. To our knowledge, it is the first application of 2 PM to explore the vascular effects of nsPEFs and to use imaging in this convenient model system that encompasses some of the vascular complexity of *in vivo* murine and human tumors. The resolution of our time-lapse intravital imaging approach allowed us to clearly establish that nsPEFs target neovascular capillary networks in tumors.

The decrease in perfusion observed in glioblastoma occurred after a single 10 ns PEF treatment and was similar to that previously measured with Doppler ultrasound in a murine model of melanoma treated with 300 ns pulses at the same electric field intensity (~45 kV/cm)^8^,^23^. Our observation that a 10 ns PEF was sufficient to significantly decrease tumor blood flow was surprising and the fact that this occurred with a single pulse rules out potential thermal mechanisms associated with cumulative Joule heating from pulse repetition. We have recently used fluorescence imaging to measure that the thermal impact of a single 10 ns pulse is less than 0.5 °C^24^. Similarly, Pliquet and colleagues have shown that nsPEF treatment effects *in vivo* are not hyperthermic at low repetition rates by measuring temperature with thermo-sensitive liquid crystals^25^. The effect of nsPEFs on vasculature is thus non-thermal.

Multiphoton imaging of tumor organoids grown in the CAM provided excellent spatial resolution with temporal sampling over minutes to 24 h. However, this method was not suited to the long term observations possible in rodent xenograft models that have shown complete tumor destruction hundreds of days after a single nsPEF treatment. In our system, it was visually apparent that the decrease in perfusion observed in nsPEF treated glioblastoma did not return at 24 h; but, it was not possible to follow the fate of the tumor beyond this timeframe due to technical constraints associated with fluorescent labelling and the development of the embryo. Intravital imaging in the CAM therefore offers a means to study early events following nsPEF treatment at the cellular level in the microenvironment of 3D tumors over short timescales (minutes to tens of hours).

In our experiments, the endogenous vasculature of the CAM was also influenced by the application of a single 10 ns electric pulse. Effects on larger vessels were reversible and were manifest as slow, transient contractions in arteriole and venule diameter. In contrast, capillaries at the CAM surface irreversibly collapsed with or without extravasation. Perfusion did not recover in the time scale of our observations (24 h). An extensive comparison of the relative sensitivity of tumor capillaries vs. normal microvasculature was not performed with respect to the electric field strength, given the complexity of the vascular network. Whether this complexity was also the source of the variable responses of CAM vasculature (bleeding versus non-bleeding) will be the subject of future investigations. Numerical tools based on FDTD calculations are currently under development by our group to extract vascular geometry from multiphoton imaging stacks and establish macroscopic dosimetry to consider the role of vessel density and other factors on tissue level nsPEF effects.

The decrease in vascular perfusion caused by nsPEFs was similar to that previously seen by other groups investigating the effects of electrochemotherapy using pulses of longer duration (100 μs). In this case, electric pulses were shown to influence the perfusion of mouse fibrosarcoma tumors^26^, mouse skin^27^, mouse hindlimb muscle^28^ and rabbit organs^29^. Our results suggest that there may be a general effect of electrical stimulation on tissue perfusion that extends down to the nanosecond regime. Others investigators have also used intravital imaging to show that μs-electric pulses arrests vascular perfusion, causing a ‘vascular-lock’, with subsequent increases in capillary permeability^27^,^30^. The predominance of the observed effect of nsPEFs on capillaries, and absence of innervation in the CAM blood network, allows us to rule out neurovascular mechanisms and points towards direct effects on endothelia or their junctions^31^. Such immediate effects on endothelial cells are expected as mathematical models predict that vessel walls experience a 40% greater electric field than the surrounding tumor^32^ and experimental observations of endothelial swelling and apoptosis support these calculations. On the contrary, studies of irreversible electroporation directly applied to externalized arteries have shown no immediate effect on endothelia, but significant long-term decreases in smooth muscle cells in the vessel wall^33^. It is important to also note that irreversible electroporation is typically associated with necrosis, whilst nsPEFs lead to apoptosis.

In contrast to the re-perfusion that occurs in tissue treated with 100 μs electric pulses, in our hands, blood flow did not return to tumor microvasculature or endogenous capillary beds in samples treated with nsPEFs. This is in agreement with the irreversible collapse of tumor perfusion observed in nsPEF treated melanoma and confirmed by histological sections^8^,^23^. Further multiphoton imaging experiments are needed in the >24 h time scale to confirm the persistence of our findings from the CAM model and determine by histological means whether these effects resemble vascular ablation with subsequent endothelial necrosis or apoptosis.

The changes in capillary perfusion and permeability that we have observed following nsPEF delivery appear similar to those that occur in endothelial barrier dysfunction (EBD). Mechanistically, EBD has been shown to involve cytoskeleton reorganization and actomyosin contraction that is mediated by Ca^2+^-dependent myosin light chain kinases and Rho associated kinases^34^,^35^. Another important factor in EBD is dynamics of microtubule (MT) assembly and disassembly^36^. In this case, the disruption of the MT network leads to an increase in transendothelial permeability. Indeed, in the CAM model MT depolymerization collapses capillary structures^37^. Given that nsPEFs have been shown to cause increases in cytosolic Ca^2^+ 38 and disrupt cytoskeleton organization^39^,^40^,^41^,^42^, it would stand to reason that the capillary collapse we observed in CAM and tumors could be mediated by these effects. One would expect tumor vasculature to be more sensitive to MT disruption due to their abnormal organization^43^. Future experiments should investigate the potential role of endothelial barrier cytoskeleton in nsPEF vascular effects and determine the comparative sensitivity of tumor vasculature versus normal tissue, which was beyond the scope of the current study.

The majority of investigations on nsPEF effects have been carried out *in vitro* and further study of the mechanisms involved *in vivo* will facilitate the application of this treatment to other types of solid tumors in humans. The CAM tumor xenograft system is less expensive when compared with mammalian models that are logistically more difficult and costly. This convenience is offset by the short temporal window for tumor growth to observe treatment effects. Our CAM approach consisted of imaging fluorescent tumor xenografts a few days after implantation, when tumors supplied with vessels of CAM origin clearly became visible. In addition to proliferating at the place of implantation, tumor cells in the CAM eventually metastasize to internal organs of the embryo and are easily identifiable^44^. By using this imaging approach, it will be possible to observe the consequence of collapsing tumor microvasculature on calcium and other early signaling pathways by using tumor lines expressing genetically encoded functional indicators. The addition of bioluminescence tools would allow more long-term measures of apoptosis, necrosis and tumor regression. Biophotonics tools can therefore significantly contribute to the fundamental understanding of nsPEF effects in complex tissue environments with the ultimate goal of developing human cancer therapeutics that are painless, without side effects and highly targetable in tissue.

Finally, the risk of applying electric pulses in the nanosecond domain to treat tumors embedded in an electrically excitable tissue like the brain has not yet been investigated. This is an important consideration as the pulse durations associated with electroporation-based technologies (μs to ms)^2^ would undoubtedly influence neuronal activity as they are temporally similar and more intense to those already used in clinical neurostimulation^45^,^46^. Multiphoton imaging of glioblastoma is also feasible in an orthotopic murine model^22^,^47^ and experiments are currently underway in our group to determine whether it is indeed possible to apply nsPEFs *in vivo* to collapse tumor vasculature without damaging nearby electrically excitable tissue comprised of neurons.

## Methods

### Tumor cell lines and culture

Human U87-MG glioblastoma cells (ATCC HTB-14) were modified by lentiviral infection to stably express fLuc2 and eGFP (U87-fLuc2/eGFP, maximum excitation λ = 488 nm, maximum emission λ = 509 nm) under the control of the CMV promoter (kindly provided by Dr. S.A. Collins from Department of Medicine-Digestive Diseases, UCLA, USA)^48^. They were grown at 37 °C in a 5% CO_2_ humidified atmosphere, in Modified Essential Medium EARLES (10370–047, Gibco, France) supplemented with 10% fetal bovine serum (10500–064, Gibco, France), 0.2% Glucose (19002–013, Gibco, France), 2 mM L-glutamine (X0550, Dominique Dutscher, France) and 100 U/ml penicillin and 100 μg/ml streptomycin (15140155, Gibco, France). The viability of U87 cells was greater than 90% as determined by visual counts using Trypan Blue Dye Exclusion on a Malassez cell, or by analysis with the Muse^®^ Cell Analyzer (MCH100102, Muse Count & Viability Assay Kit, Millipore).

### CAM xenograft assays

The chorio-allantoic membrane (CAM) assay was developed with quail eggs and adapted from previously described protocols^20^,^49^. Fertilized eggs of Japanese quail (*Coturnix coturnix japonica*) were obtained from a local supplier (Japocaille, Saint Euphrône, France) and incubated in a forced-air incubator on trays with an automatic rotator that turned eggs twice daily (39.5 °C, humidity 60%). On ED3.5 (the embryonic day when eggs were set in the incubator was counted as ED0), they were gently cleaned with 70% ethanol, cracked in a sterile laminar flow hood into 20 cm^2^ plastic weigh boats, covered with plastic wrap, and transferred to a standard humidified incubator at 37 °C as shell less culture (Fig. 1a). Dead or infected embryos were eliminated daily to avoid further contaminations. At ED7.5, U87 cells were rinsed in Modified Essential Medium EARLES (10370–047, Gibco, France) and supplemented with 0.2% Glucose (19002–013, Gibco, France) and 2 mM L-glutamine (X0550, Dominique Dutscher, France) and a suspension of (2.10^5^ cells in 5 μl of medium) was deposited on the intact CAM, distal from the embryo and major blood vessels. The embryo was maintained in the incubator until the shape of the tumor was visible and a clear vascularization could be observed (inspected with a stereomicroscope), usually at 48 h post-implantation. Multiphoton intravital imaging was performed and nsPEF delivered when tumors reached approximately 0.5–2 mm in diameter.

### nsPEF Exposure System

The nsPEF exposure setup (Fig. 1b) comprised of an nsPEF generator, a high-speed digital oscilloscope, a high-voltage attenuation measurement device (tap-off) and an electrode-based delivery system at the microscope stage.

Electrodes were made of stainless steel hypodermic tubing (18G, 1.27 mm external diameter) connected to a BNC connector (Amphenol RF, USA) with a 50 Ω resistor (RR5025, Vishay, USA). The hypodermic tubes were flattened and the edges smoothed in order to maximize surface contact without damaging the CAM surface. The resistor was placed in parallel with the electrodes to ensure impedance matching between the transmission lines and the generator^50^. The electrodes were slightly bent in order to facilitate their positioning on the biological sample. The exposure channel formed by the electrodes was 3 mm long, 0.5 mm high and 1.2 mm in length. To ensure electrical isolation and mechanical stability, rubber insulator covers were placed upstream to electrode tips (Fig. 1c). The delivery system was inserted across the *ex ovo* biological sample under the microscope objective.

The nsPEF generator (FPG 10-1NM-T, FID Technology, Germany) had an output impedance of 50 Ω and it delivered 10 ns duration pulses with adjustable amplitude between 4.5 kV and 10 kV and rise-times around 5 ns. A 1 GHz oscilloscope (DPO 4104, Tektronix, USA) was connected to the 40 dB attenuation tap-off using a 30 dB attenuator to display the time-domain measurements of the delivered electrical pulses. The tap-off (245 NMFFP-100, Barth Electronics Technology, USA) was connected to the nsPEF generator, the oscilloscope and the delivery system using three transmission lines (RG 214 cable) of different lengths. The tap-off and the transmission lines allowed the extraction of the delivered pulses from the incident and reflected pulses for monitoring the applied pulses^51^,^52^,^53^ (Fig. 1b). An example of a delivered pulse is shown in Fig. 2a.

The dielectric properties (i.e. the relative permittivity ε_r_, and the electrical conductivity σ) of the *ex ovo* biological sample were measured with a dielectric probe (85070E Dielectric probe kit, Agilent, USA).

### Dosimetry - Numerical simulations

The numerical dosimetry of the nsPEF exposure setup was performed using numerical modeling and full-wave 3D simulations based on a discretization of Maxwell’s equations in integral form. This numerical tool allowed rigorous analysis of complex and inhomogeneous structures. Dosimetric computations of the delivery system and the exposed sample were made in terms of reflection coefficient and the spatial distribution of the electric field obtained from simulations.

The delivery system shown in Fig. 1 was modeled and simulated including the plastic weigh boat containing the biological sample. All the dielectric materials were taken into account in the simulations through their electromagnetic macroscopic properties, i.e. relative permittivity and electrical conductivity. The relative permittivities and electrical conductivities are provided in Fig. 2b. The metallic parts of electrodes, BNC inner and outer rings were considered as perfect electric conductors. The electromagnetic feed was placed at the input of the delivery system (BNC connector level) as a 50 Ω localized source. A 50 Ω resistor was also placed across the electrodes. For computations, the delivery system was spatially discretized with a non-uniform mesh. The mesh steps were 50 ×50 ×50 μm^3^ between the electrodes exposure channel and 200 × 200 × 200 μm^3^ for the other parts of the structure.

To determine the efficiency of the energy transfer between the generator and the delivery system, reflection coefficient (S_11_) evaluation was carried out through measurements and simulations (Supplementary Fig. 3). The lower the reflection coefficient value, the better the energy transfers between the generator and the delivery system. Reflection coefficients of less than −13 dB over the 0–500 MHz frequency bandwidth (without biological solution) and less than −10 dB over the 0–200 MHz frequency bandwidth (with biological solution) were obtained corresponding to a good impedance matching and energy transfer (SFig. 3).

### Multiphoton imaging system and time-resolved fluorescence measurements

The embryos were positioned on a heated microscope stage of a customized multiphoton microscope BX61WI/FV1200MPE (Olympus) coupled with a tunable femtosecond Ti:Sapphire pulsed laser (Chameleon Ultra II, Coherent, Glasgow, UK) for the excitation. A set of mirrors (PF10-03-P01, Thorlabs) and a beam expander (BE02-05-B, Thorlabs) adaptated the beam size and axis into a 25X immersion objective (XLPLN25XWMP, 1.05 numerical aperture, 2 mm working distance, Olympus). The average power was adjusted with a half wave plate and a polarization cube (WPH10M-830, CM05-PBS202, Thorlabs). The electrodes were positioned on both sides of the region of interest, and immersed in the CAM surface under the water immersion microscope objective with Live Cell Imaging Solution (A14291DJ, Molecular Probes, France) composed of 140 mM NaCl, 25 mM KCl, 1.8 mM CaCl_2_, 1 mM MgCl_2_, 20 mM HEPES at pH7.4 (Fig. 1b). In the case of control experiments, electrodes were also positioned to the sample but the nanosecond pulse generator was not activated. To visualize microcirculation, a vein was cannulated using a glass capillary needle (1B150F-4, WPI, UK) made with a vertical microelectrode puller (P-1000, Sutter Instruments, Novato, CA, USA) and connected to a silicon tube filled with 20 μl of 10 mg/ml of a Rhodamine dye solution dissolved in PBS 1X (Rhodamine B isothiocyanate dextran 70K, R9379, Sigma Aldrich, France, maximum excitation λ = 554 nm, maximum emission λ = 627 nm). 3D images stacks were collected at 2 μm intervals using multiphoton excitation at 860 nm every 5 min with FluoView FV1200 software (v4.1.1.5, Olympus). Excitation light was separated from the emitted fluorescence with a dichroic mirror (<650 nm). The different components of the light emitted from the sample were separated using a further dichroic mirror (570 nm) distributing the green and red fluorescence to two photomultiplier tubes (PMT voltage 350 V and PMT voltage 444 V) with fluorophore specific emission filters (BA 575–630 for Rhodamine B, GFP 495–540 for GFP).

### Image acquisition and processing

Bright field images were obtained on a MVX10 Research MacroView Microscope (Olympus) with a DP73 camera at 16-bits (Olympus). Multiphoton fluorescence images were acquired in frame scanning mode with 12 bits per pixel, x = 256, y = 256. The z stack depth was variable between 200 and 300 samples depending on the experiment (each z step = 2 μm) and thickness of the tumor. Image stacks were sampled at 5 minute intervals due to the time required to acquire multiple planes in the z-axis. Images stacks were segmented and rendered to 3D volumes. 3D volumes and fluorescence intensity were automatically quantified by Imaris software (Bitplane AG, Switzerland). Vessel diameters were measured manually using the «Measuring Distances» tool in Imaris, by choosing delimiting points on each side of the vessel walls. Images were background corrected and median filtered equally across treated samples and controls. Figures were prepared using Adobe Photoshop CS6 (version 13.0) after conversion of RGB or grayscale images.

### Statistical analysis

Statistical analysis was performed with OriginPro 2016 (Ritme, France). The relative changes in blood volume (ΔV/V0), fluorescence (ΔF/F0) and diameter (Δd/d0) were calculated as percentages of the initial measure (V0, F0 and d0, at t = 0 min). All data were first tested to see whether they were normally distributed (Chen-Shapiro, Shapiro-Wilk, Kolmogorov-Smirnov tests). Data were analyzed with a two-way repeated measure of analysis of variance (ANOVA), with post-hoc tests to locate the source of significant differences using Bonferroni or Sidak corrections for multiple comparisons. Kruskal-Wallis non parametric tests were used where data was not normally distributed. The results are presented as means with standard error (±SEM). All tests used were two-sided. Asterisks in the figures indicate statistically significant differences compared to controls where the probability of falsely rejecting the null hypothesis was less than 5% (p < 0.05).

## Additional Information

**How to cite this article**: Bardet, S. M. *et al*. Multiphoton imaging reveals that nanosecond pulsed electric fields collapse tumor and normal vascular perfusion in human glioblastoma xenografts. *Sci. Rep*. **6**, 34443; doi: 10.1038/srep34443 (2016).

## Supplementary Material

Supplementary Information

Supplementary Video S1

Supplementary Video S2

Supplementary Video S3

Supplementary Video S4

Supplementary Video S5

Supplementary Video S6

Supplementary Video S7

Supplementary Video S8

Supplementary Video S9

Supplementary Video S10

Supplementary Video S11
