# Supplementary material for: Multiphoton imaging reveals that nanosecond pulsed electric fields collapse tumor and normal vascular perfusion in human glioblastoma xenografts
Source: Sci Rep. 2016 Oct 4;6:34443. doi: 10.1038/srep34443 (PMC5048165; doi:10.1038/srep34443)
Supplement: Supplementary Information [file srep34443-s1.pdf]

Multiphoton imaging reveals that nanosecond pulsed electric fields collapse tumor and normal vascular perfusion in human glioblastoma xenografts.

Sylvia M Bardet[1], Lynn Carr[1], Malak Soueid[1], Delia Arnaud-Cormos[1], Philippe Leveque[1] & Rodney P O'Connor[1]

[1] XLIM Research Institute, UMR CNRS No 7252, University of Limoges, Faculty of Science and Techniques, 123 Avenue Albert Thomas, 87060 Limoges, France.

Corresponding author: Rodney P. O'Connor rodney.oconnor@xlim.fr

SUPPLEMENTARY FIGURES

Supplementary figure 1: The nanosecond pulsed electric field effects on capillary perfusion in the CAM persist at 12h. CAM vascularization was visualized with bright field microscopy before nsPEF application **(a)**, 5 min after **(b),** and at 12 hours **(c)**. Electrodes site were drawn on **(a)** with dotted rectangles. The circular zone (1) on **(b)** highlights the clear loss of perfusion of the treated zone (large vessels and capillaries) 5 min after nsPEF treatment. Circle 2 in **(c)** shows an example zone where perfusion did not return in capillaries, compared with another zone (c, circle 3) where vascularization recovered after 12 hours. Scale bar in (c) = 2 mm applies to all images.

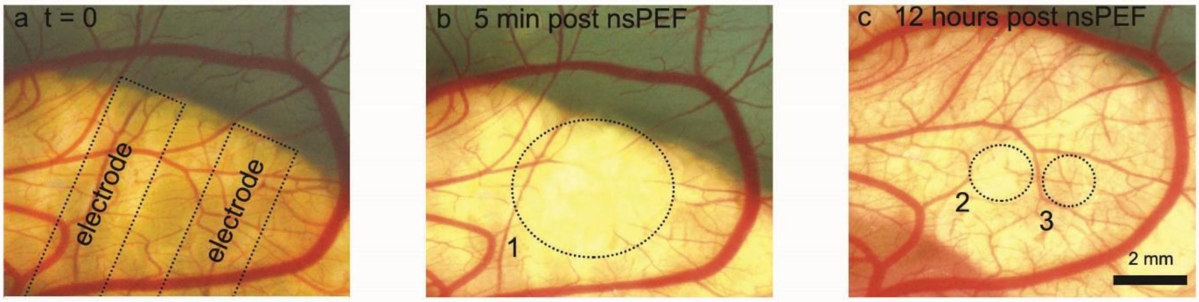

Supplementary figure 2: The dose-response relationship for the vascular effects of nsPEF was investigated with respect to electric field intensity. A total of 18 independent CAM samples were injected with Rhodamine B-dextran 70k and treated with a single nsPEF at a range of electric field intensities including 0 kV/cm (control condition with the same placement of electrodes, n=4), 10.6 kV/cm (n=2), 16.5 kV/cm (n=2), 22.5 kV/cm (n=3), 34 kV/cm (n=3) and 44kV/cm (n=4). Vessel diameter was measured and the peak decrease in vessel diameter followed a sigmoidal trend with increasing electric field intensity, as shown by the dose-response curve fit (R-Square=0.99).

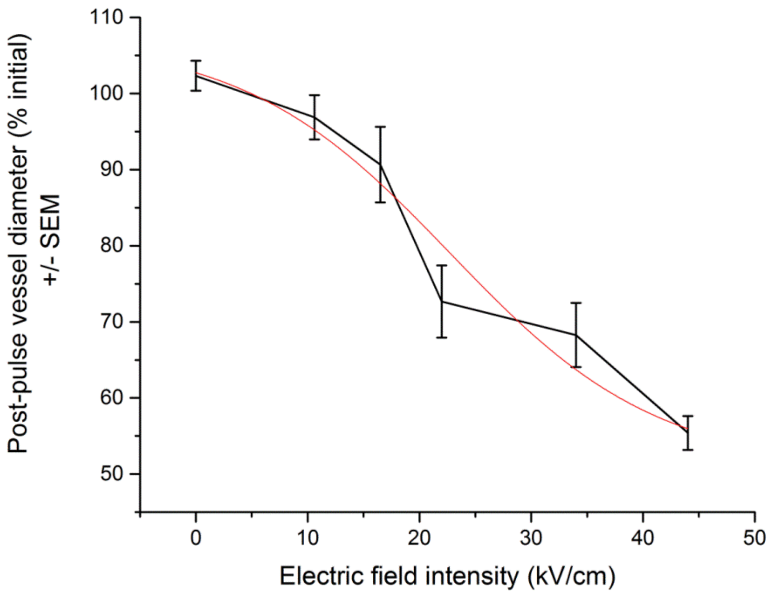

Supplementary figure 3: The measured and simulated reflection coefficient curves of the nsPEF delivery electrodes. To determine the efficiency of the energy transfer between the generator and the delivery system, reflection coefficient (S_{11}) evaluation was carried out through measurements and simulations. Reflection coefficients of less than -13 dB over the 0-500 MHz frequency bandwidth (without biological solution, dotted lines) and less than -10 dB over the 0-200 MHz frequency bandwidth (with biological solution, solid lines) were obtained corresponding to a good impedance matching and energy transfer. It can be noticed that both measured and simulated results are in good agreement with each other.

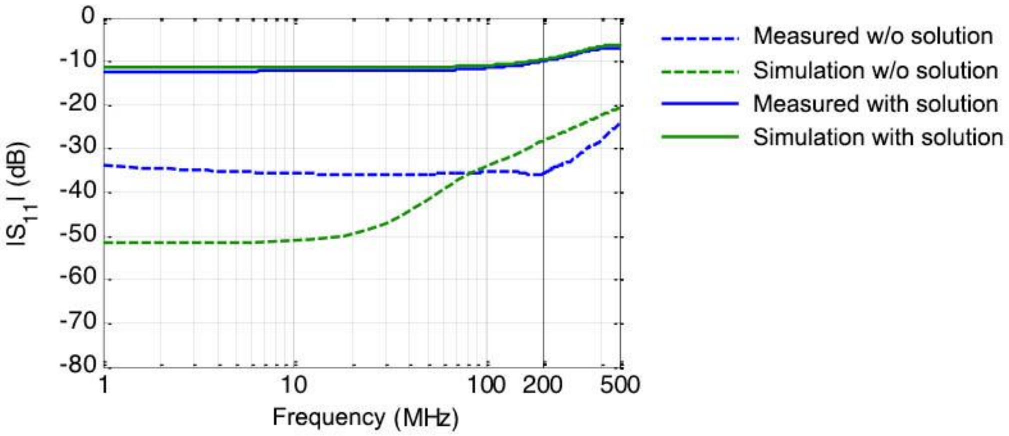

Supplementary video 1 :

Multiphoton imaging of quail CAM vasculature visualized in a 3D rotating movie showing intravascular Rhodamine dextran labeled capillaries and vessels in a field of 500*500*200 µm (case 1).

Supplementary video 2 :

Multiphoton imaging of quail CAM vasculature visualized in a 3D rotating movie showing intravascular Rhodamine dextran labeled capillaries and vessels in a field of 500*500*400 µm (case 2).

Supplementary video 3 :

Multiphoton imaging of quail CAM vasculature visualized in a 4D movie (3D over time) showing intravascular Rhodamine dextran labeled capillaries and vessels in a field view of 500*500*400 µm (case 2) that was pulsed at t=6 min with a single 10 ns PEF.

Supplementary video 4 :

Multiphoton imaging of quail CAM vasculature visualized in a 3D rotating movie showing intravascular Rhodamine dextran labeled capillaries and vessels in a field of 500*500*350µm (case 3).

Supplementary video 5 :

Multiphoton imaging of quail CAM vasculature visualized in a 4D movie (3D over time) showing intravascular Rhodamine dextran labeled capillaries and vessels in a field view of 500*500*350 µm (case 3) that was pulsed at t=6 min with a single 10 ns PEF and displayed extravascular fluorescence.

Supplementary video 6 :

Multiphoton imaging of quail CAM vasculature visualized in a 3D rotating movie showing intravascular Rhodamine dextran labeled capillaries and vessels in a field of 500*500*500µm (case 4).

Supplementary video 7 :

Multiphoton imaging of quail CAM vasculature visualized in a 4D movie (3D over time) showing intravascular Rhodamine dextran labeled capillaries and vessels in a field view of 500*500*500 µm (case 4) that was pulsed at t=6 min with a single 10 ns PEF and displayed extravascular fluorescence.

Supplementary video 8 :

Intravascular Rhodamine B-dextran and GFP-U87 grafted on CAM were observed with multiphoton imaging in a series of image sections of the tumoral spheroid over 500*500*250 µm (case 5).

Supplementary video 9 :

Intravascular Rhodamine B-dextran and GFP-U87 grafted on CAM were observed with multiphoton imaging and shown in a 3D rotating movie over 500*500*200 µm (case 6).

Supplementary video 10 :

Intravascular Rhodamine B-dextran and GFP-U87 grafted on CAM were observed with multiphoton imaging and shown in a 3D rotating movie over 500*500*200 µm (case 7).

Supplementary video 11 :

Intravascular Rhodamine B-dextran and GFP-U87 migrating cells on CAM were observed with multiphoton imaging and shown in a 3D rotating movie over 500*500*200 µm (case 8).
